# Editorial: National colorectal cancer awareness month 2025: current progress and future prospects on colorectal cancer prevention, diagnosis and treatment

**DOI:** 10.3389/fonc.2026.1915773

**Published:** 2026-07-02

**Authors:** Alessandro Passardi, David Gibbons, Rocco Ricciardi, Simona Gurzu

**Affiliations:** 1 Department of Medical Oncology, IRCCS Istituto Romagnolo per lo Studio dei Tumori (IRST) “Dino Amadori”, Meldola, Italy; 2 Department of Pathology, St Vincent’s University Hospital, Dublin, Ireland; 3 School of Medicine and Medical Sciences, University College Dublin, Dublin, Ireland; 4 Massachusetts General Hospital, Harvard Medical School, Boston, MA, United States; 5 George Emil Palade University of Medicine, Pharmacy, Sciences and Technology of Târgu Mureş, Târgu Mures, Romania

**Keywords:** artificial intelligence, colorectal cancer, molecular biomarkers, precision medicine, prevention, screening, survivorship

The Research Topic *“National Colorectal Cancer Awareness Month 2025: Current Progress and Future Prospects on Colorectal Cancer Prevention, Diagnosis and Treatment”* was conceived with the aim of promoting scientific discussion and increasing awareness around one of the leading causes of cancer-related morbidity and mortality worldwide. The remarkable response from the scientific community, resulting in 29 accepted manuscripts and outstanding dissemination metrics, confirms the growing global interest in colorectal cancer (CRC) prevention, early diagnosis, translational research, and personalized treatment strategies.

The articles collected in this Research Topic provide a broad and multidisciplinary overview of the rapidly evolving CRC landscape, spanning epidemiology, screening, molecular biology, prognostic modeling, surgical innovation, systemic therapies, survivorship, and healthcare disparities. Together, these contributions highlight how CRC management is becoming increasingly precision-oriented, technology-driven, and patient-centered.

A major theme emerging from this Research Topic is the central role of prevention and screening. Several studies investigated the global burden of CRC and the contribution of modifiable risk factors (Zeng et al.), including dietary habits and metabolic disorders such as hyperglycemia (Chen et al.; Li et al.), as well as age-related clinicopathological patterns (Liu et al.). These analyzes reinforce the urgent need for prevention-oriented public health strategies and equitable access to screening programs. Additional studies explored economic burden and healthcare resource utilization in CRC management (Hu et al.), further emphasizing the system-level challenges associated with this disease.

In parallel, important advances in CRC screening technologies were highlighted. Innovative non-invasive approaches, including stool DNA methylation assays (Li et al.) and fecal biomarkers such as calprotectin (Jing et al.), demonstrated promising diagnostic potential. Moreover, a comprehensive framework on emerging CRC screening technologies underscored the rapidly evolving landscape of early detection strategies (Sapienza et al.). Barriers to screening implementation in real-world settings were also explored, highlighting persistent gaps in access and uptake (Busbait).

Another key area concerns prognostic stratification and personalized oncology. Several studies proposed novel clinicopathological models, nomograms, inflammatory biomarkers, and machine-learning algorithms to improve risk prediction and survival estimation in CRC patients (Xie et al.; Zhang et al.; Cao et al.; El Muhtaseb et al.). These contributions reflect the ongoing integration of computational methods and real-world evidence into clinical oncology. Tumor deposit-based staging refinements further emphasize the need to move beyond traditional TNM classification alone (Zhang et al.).

The molecular characterization of CRC also emerged as an important topic. Studies addressing mismatch repair deficiency and lymph node metastasis risk (He et al.), as well as analyzes of metastatic dissemination patterns such as lung metastasis (Zhang et al.), contribute to a more biologically driven understanding of disease behavior. Translational research into regulatory pathways and experimental mechanisms, including TBX5-related signaling and traditional medicine-derived compounds, further expands the spectrum of potential therapeutic targets (Li et al.).

Therapeutic innovation remains a cornerstone of modern CRC care. Surgical and perioperative advances were represented by studies evaluating minimally invasive colorectal resection techniques (Li et al.), optimization of pneumoperitoneum pressure and postoperative recovery (Lu et al.), and lymph node retrieval enhancement strategies using targeted dye infusion (Lu et al.). Locoregional treatment strategies for metastatic disease were also addressed, including combined thermal ablation and liver resection approaches for colorectal liver metastases (Zhao et al.).

Systemic therapy continues to evolve rapidly. Neoadjuvant strategies in pMMR/MSS rectal cancer are reshaping therapeutic paradigms in selected patient populations (Meng et al.). Furthermore, bibliometric analysis of antiangiogenic therapy highlights sustained innovation and ongoing refinement of targeted approaches in CRC treatment (Farooq et al.). A real-world analysis of sequential regorafenib and trifluridine/tipiracil-based regimens in metastatic CRC provides important insights into treatment sequencing in clinical practice (Hou et al.).

An additional strength of this Research Topic is the attention dedicated to survivorship and quality of life. Multiple contributions evaluated symptom burden, postoperative functional outcomes, chemotherapy-induced toxicity, and patient-reported outcomes in CRC survivors (Bekaii-Saab et al.; Adli et al.; Ding et al.; Xiao et al.). These findings reinforce the importance of integrating quality-of-life measures into routine oncologic care, alongside traditional survival endpoints.

Overall, this Research Topic reflects both the remarkable progress achieved in CRC research and the ongoing challenges that remain. Earlier diagnosis, improved molecular characterization, integration of artificial intelligence, liquid biopsy technologies, immunotherapy, and minimally invasive surgical techniques are all expected to shape the future of CRC management. However, translating these advances into meaningful clinical benefit for all patients will require continued collaboration among clinicians, researchers, institutions, and policymakers. Furthermore, recent analyzes of CRC mortality trends in the United States remind us that, despite significant advances, continued efforts in prevention, screening, and equitable access to care remain essential to further reduce CRC-related mortality (Feng et al.).

As Guest Editors, we sincerely thank all authors, reviewers, and editorial staff who contributed to the success of this Research Topic. Their work has created a valuable multidisciplinary platform that not only celebrates National Colorectal Cancer Awareness Month but also contributes meaningfully to advancing CRC research and improving patient care worldwide.

